# Affinity of Keratin Peptides for Cellulose and Lignin: A Fundamental Study toward Advanced Bio-Based Materials

**DOI:** 10.1021/acs.langmuir.2c01140

**Published:** 2022-08-05

**Authors:** Emmi-Maria Nuutinen, Juan José Valle-Delgado, Miriam Kellock, Muhammad Farooq, Monika Österberg

**Affiliations:** † Sustainable products and materials, VTT, Technical Research Centre of Finland, Tietotie 2, FI-02044 Espoo, Finland; ‡ School of Chemical Engineering, Department of Bioproducts and Biosystems, Aalto University, 02150 Espoo, Finland

## Abstract

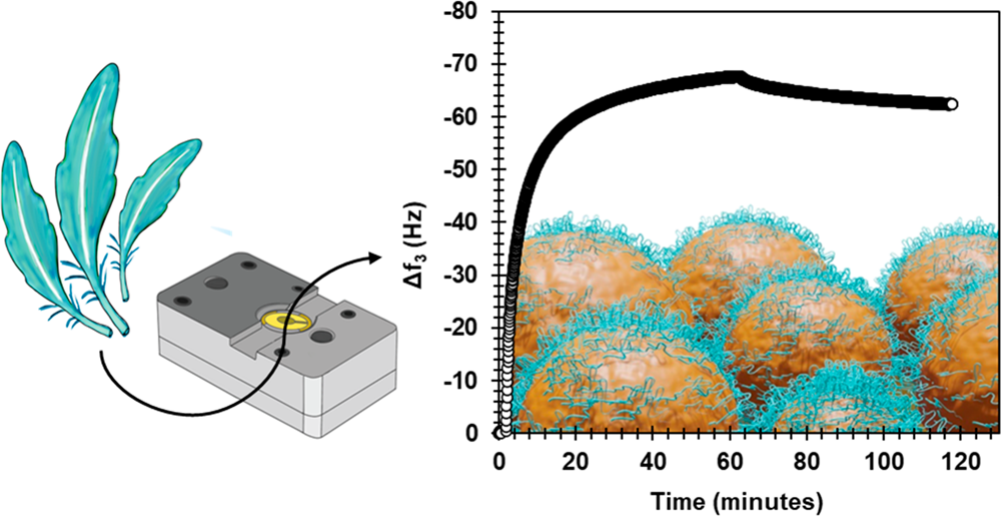

Keratin is a potential raw material to meet the growing demand for bio-based materials with special properties. Keratin can be obtained from feathers, a by-product from the poultry industry. One approach for keratin valorization is to use the protein to improve the properties of already existing cellulose and lignin-based materials to meet the requirements for replacing fossil-based plastics. To ensure a successful combination of keratin with lignocellulosic building blocks, keratin must have an affinity to these substrates. Hence, we used quartz crystal microbalance with a dissipation monitoring (QCM-D) technique to get a detailed understanding of the adsorption of keratin peptides onto lignocellulosic substrates and how the morphology of the substrate, pH, ionic strength, and keratin properties affected the adsorption. Keratin was fractionated from feathers with a scalable and environmentally friendly deep eutectic solvent process. The keratin fraction used in the adsorption studies consisted of different sized keratin peptides (about 1–4 kDa), which had adopted a random coil conformation as observed by circular dichroism (CD). Measuring keratin adsorption to different lignocellulosic substrates by QCM-D revealed a significant affinity of keratin peptides for lignin, both as smooth films and in the form of nanoparticles but only a weak interaction between cellulose and keratin. Systematic evaluation of the effect of surface, media, and protein properties enabled us to obtain a deeper understanding of the driving force for adsorption. Both the structure and size of the keratin peptides appeared to play an important role in its adsorption. The keratin–lignin combination is an attractive option for advanced material applications. For improved adsorption on cellulose, modifications of either keratin or cellulose would be required.

## Introduction

Resource sufficiency and climate change are the big challenges of our century, and various bio-based material solutions are possible ways to address these. The suitability of lignocellulosic biomass in material applications is already well recognized, and there is active research ongoing to find new lignocellulosic materials solutions.^1−5^ Besides lignocellulose, the most abundant renewable biomass composing of cellulose, hemicellulose, and lignin, there are also other possibilities to respond to these challenges, one of them being proteins. Especially, structural proteins have been identified as a potential raw material for material applications.^6,7^

Structural proteins including collagens, keratins, resilin, elastins, and silks differ from functional proteins such as enzymes and antibodies.^6^ One of the typical characteristics of structural proteins is that they have an amino acid sequence that repeats and forms highly ordered secondary structures.^6,7^ Besides their natural abundance, structural proteins can be considered biodegradable and biocompatible, and their specific mechanical, optical, electrical, chemical, biological, and thermal properties are interesting when it comes to material applications, especially in the field of biomedical applications such as biosensors or tissue regeneration.^7^ However, this field requires more studies to establish the optimal use of proteins in material applications.

Keratin is a significantly underutilized protein source. It is the main component of wool, hair, nails, hooves, feathers, and horns.^8^ Especially, feather keratin, a side-stream from the poultry industry, is currently mostly buried in the landfills, burned, or used as a poorly digestible feed. Feathers contain about 90 % keratin making them an excellent protein source.^9^ Due to the complex structure of feather keratin, its valorization in applications requires conversion of the feather keratin into a more utilizable form. Dissolution and regeneration have been identified as a feasible process to obtain keratin in a processable form for different applications.^9^ Recently, it was found that a deep eutectic solvent (DES), an environmentally friendly and inexpensive solvent, was able to dissolve feather keratin.^10^ However, the DES-treated keratin did not meet the mechanical requirements to be used in film applications.^11^ Previous studies show that when feathers are processed with DES, ionic liquid, or *N*-methylmorpholine *N*-oxide, the keratin loses its ordered structure.^10,12,13^ Moreover, in these processes, keratin degrades into different sized fragments and even into small peptides and amino acids^10,13^ leading to the loss of its mechanical properties. However, our previous work showed that together with a plasticizer, the low molecular weight keratin fraction was able to form a dense and uniform film network with decreased water vapor permeability.^11^ Hence, we speculate that together with lignocellulosic building blocks that provide the mechanical strength, the acquired keratin fraction having different amino acids with different chemical structures could provide properties lacking from these materials. Keratin could make the lignocellulosic materials more suitable for example in medical, cosmetics, electronics, agriculture, textile, and composite industries.^9^ Keratin has shown potential for example in wound healing,^14^ tissue engineering,^15^ controlled drug release,^16^ flame retardancy,^17,18^ skin hydration and elasticity improvement,^19^ and electronic materials^20^ and as a bioadsorbent for dye,^21^ metal ions,^22^ and oil.^23^

Some attempts have already been made to combine feather keratin with cellulose^24−26^ and lignin.^27^ Nevertheless, we are lacking understanding of the interactions between keratin and cellulose and keratin and lignin. Understanding of interactions is crucial in order to successfully combine these materials in applications.

This work aims to address the gap in our understanding of the interaction of keratin with lignocellulosic building blocks using well-defined cellulosic and lignin thin films and fractionated feather keratin peptides. We utilized a surface sensitive method, the quartz crystal microbalance with dissipation monitoring (QCM-D), to systematically probe the adsorption behavior of keratin peptides onto thin films of cellulose, lignin, and lignin in the form of colloidal lignin particles (CLPs). By systematically addressing the effect of various factors on the adsorption behavior, we shed new light on the main driving forces for adsorption of peptides derived from structural proteins onto lignocellulosics. The gained information is expected to play a vital role in optimizing the combination of naturally occurring building blocks to design competitive bio-based products.

## Experimental Section

### Materials

Feathers were supplied by Grupo SADA (Madrid, Spain), and before their delivery, they were washed with an alkaline soap solution (95 °C for 2 h), dried (60 °C for 24 h), and then sterilized with pressurized steam (126 °C for 30 min). The absence of pathogens was confirmed with microbiological detection (ISO 16140, ISO 16140/AOAC, ISO 11290-1/A1). The used DES components were sodium acetate (NaOAc) (>99% sodium acetate anhydrous from Sigma-Aldrich) and urea (99.0–100.5% urea from Sigma-Aldrich). Trimethylsilyl cellulose (TMSC) used in the preparation of cellulose thin films was prepared by silylation of microcrystalline cellulose powder from spruce (Fluka), while softwood kraft lignin (SKL) (BioPiva 100) was used for the preparation of lignin thin films. Poly-l-lysine (PLL) solution at a concentration of 0.1% (w/v) and a molecular weight (Mw) of 150,000–300,000 g/mol and polystyrene (PS) having an Mw of 280,000 g/mol were acquired from Sigma-Aldrich. All used laboratory chemicals were of analytical grade.

### DES Fractionation of Feathers

**Figure 1:**
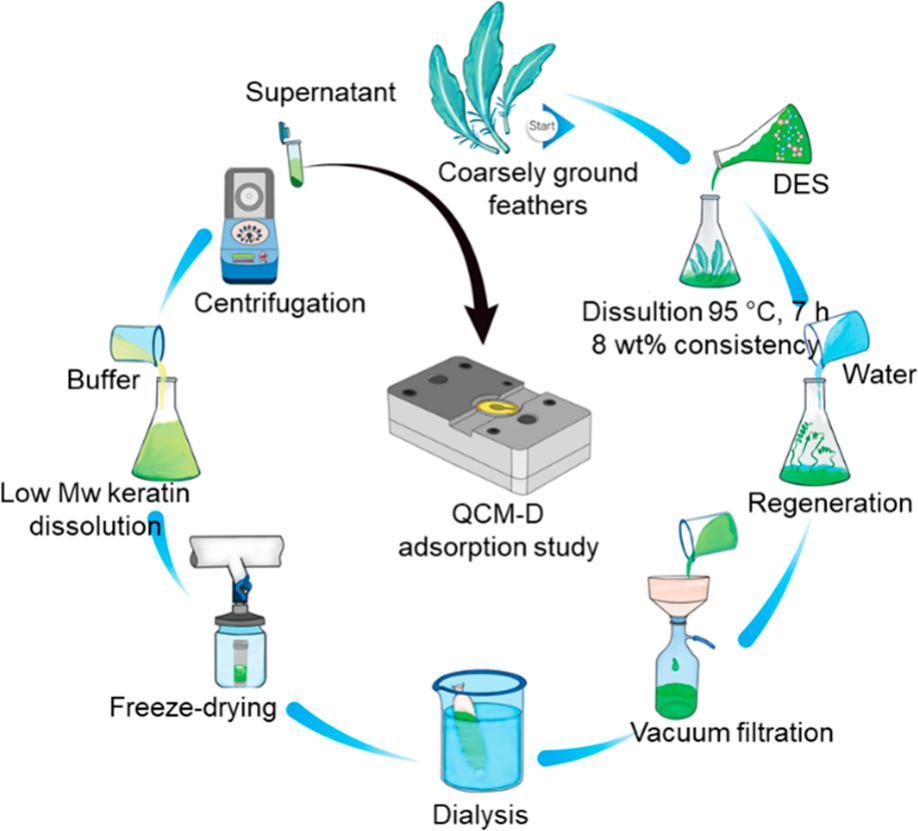
Experimental setup for the DES fractionation of keratin for the QCM-D experiments.

Feathers were ground into 2–15 mm pieces using an E-compactor (VTT, Finland) in which the feathers are pressed through a die using pan grinder rollers. DES processing was carried out as previously described^10^ with minor modifications. Compactor ground feathers (8 wt %) were added to preheated (70 °C), clear, freshly prepared solvent consisting of NaOAc and urea in the molar ratio of 1:3 and with a small addition of water (10 wt %). The keratin dissolution was carried out in a 15 L closed reactor equipped with a mixer at 95 °C for 7 h. After the dissolution, the keratin solution was added into water (20 L). This induced precipitation of keratin with higher molecular weight (Mw), while keratin with lower Mw remained dissolved. Precipitated high Mw keratin was removed from the liquid fraction by vacuum filtration, and low Mw keratin was separated from the DES components by dialysis using membranes with a 3.5 kDa cut off (Spectra/Por Standard RC Tubing, Spectrum Laboratories, CA, USA). The dialysis was stopped when the conductivity of the washing water did not change anymore. The low-Mw keratin fraction was freeze-dried; the molar mass, solubility, zeta potential, and conformation were determined; and then it was further dissolved in the buffers used for the adsorption studies. The experimental setup for the keratin fractionation is presented in Figure 1.

### Keratin Characterization

The molar mass measurements of low-Mw keratin samples were performed by size exclusion chromatography (SEC) using alkaline eluent (0.1 M NaOH). For the molar mass measurements, the keratin powder from the DES treatment was dissolved into 0.1 M NaOH and the keratin samples, which were first dissolved in sodium phosphate buffer (pH 7, 150 mM), were diluted with 1 M NaOH for the measurement concentration (1 mg/mL). For the dilution of the samples, 1 M NaOH was used due to the high acidity of the samples. In all cases, the samples were filtered (0.45 μm) before the measurement.

The SEC measurements were performed in 0.1 M NaOH eluent (pH 13, 0.5 mL/min, *T* = 25 °C) using PSS MCX 1000 & 100,000 Å columns with a pre-column. The elution curves were detected using two different detectors: a Waters 2998 Photodiode Array detector at 280 nm and a Waters 2410 RI detector. All dissolved organic material can be detected by the RI detector. The molar mass distributions (MMD) were calculated against 8× pullulan (6100–708,000 g/mol) standards typically used for the polysaccharides.
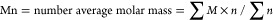
1
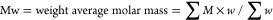
2
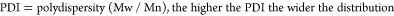
3

For the solubility and zeta potential measurements, the low Mw keratin fraction was dissolved in water (2 mg keratin/1 mL water), and HCl and NaOH were used to adjust the pH. The pH affected the keratin solubility, and insoluble keratin fractions were removed by centrifugation (3000 rpm, 5 min) after the change in pH. The amount of remaining soluble protein was measured using the Bio Rad DC Protein Assay (BSA as standard), and the zeta-potential was determined using a particle analyzer instrument (Zetasizer Nano ZS, Malvern, U.K.). An average of three replicate measurements with standard error (standard deviation/square root of total number of samples) was reported. Keratin solubility was also assessed by dissolving keratin in different buffers (2 mg keratin/1 mL buffer). The buffers used were sodium phosphate buffer (SPB) (pH 7, 50 mM, 150 mM, 500 mM), sodium acetate buffer (SAB) (pH 5, 50 mM), and McIlvaine buffer (pH 3, about 40 mM). After dissolution in buffer, the insoluble keratin fraction was separated by centrifugation and the Bio Rad DC Protein Assay was used to measure the protein content in the solution.

The circular dichroism (CD) spectra of the low-Mw keratin fraction were measured with a Chirascan CD spectrophotometer (Applied Photophysics, Leatherhead, U.K.) at different pH values and ionic strengths. Measurements were carried out in the same protein concentrations (0.1 mg/mL) by dissolving low-Mw keratin fraction in SPB buffers (pH 7, 50, 150, and 500 mM) or in water, after which the pH was adjusted to 3, 5, or 7 with HCl or NaOH. The temperature was controlled by placing a thermoprobe connected with temperature control set into the measuring cell. The CD spectra were recorded using a 1 mm cell and a bandwidth of 1 nm from 240 to 190 nm UV light. The data is expressed in terms of ellipticity (mdeg).

### Preparation of Lignocellulosic Thin Films

All the thin films were prepared onto QCM-D gold sensors (Advanced Wave Sensors S.L., gold, 5 MHz).

Cellulose thin films were prepared from trimethylsilyl cellulose (TMSC) via spin-coating. TMSC was synthesized as described earlier.^28^ Briefly, cellulose powder was first dissolved in a mixture of dimethylacetamide and lithium chloride (DMAc/LiCl) after which silylation was carried out with hexamethyldisilazane (HDMS). A total of 10 mg of solid TMSC was dissolved in 1 mL of toluene. Sensors were first spin-coated with 0.1 wt % polystyrene (PS) in toluene. Two to three droplets of PS solution were placed on UV/ozone-treated gold sensors, and the spinning was done at 4000 rpm for 30 s. The solvent was then evaporated at 60 °C for 10 min. Prior to the TMSC spin-coating, PS-coated sensors were UV/ozone-treated and wetted by applying and spinning toluene onto the sensors at 3000 rpm for 15 s. The TMSC solution was then spin-coated onto the PS coated crystals at 3000 rpm for 60 s, and the solvent was evaporated at 60 °C for 10 min. TMSC-coated sensors were regenerated back to cellulose by acid hydrolysis with hydrochloric acid vapor resulting in cellulose-coated sensors for QCM-D measurements. The PS-coated sensor was used as a control.

Lignin thin films were prepared from dissolved lignin using spin-coating as described previously.^29,30^ First, 0.5 wt % PS in toluene was spin-coated (2000 rpm, 40 s) onto UV/ozone-treated sensors. Residual solvent was evaporated at 80 °C for 30 min. Softwood kraft lignin (SKL) was dissolved in 1,4-dioxane–water (82:18 v/v) in 0.5 wt % concentration and spin-coated onto the PS coated crystals at 400 rpm for 3 s, 500 rpm for 5 s, and finally, 2000 rpm for 1 min. Four layers of SKL was spin-coated resulting in lignin-coated sensors for QCM-D measurements.

Colloidal lignin particles (CLPs) and thin films from them were prepared as described previously.^29^ Briefly, SKL was dissolved in aqueous tetrahydrofuran (THF) (75 wt %) after which particles were obtained when the lignin solution was rapidly poured into vortex-stirred deionized (DI) water followed by dialysis (a Spectra/Por 1 tubing with an MWCO of 6–8 kDa) to remove the THF. Thin films were then prepared from the obtained CLP dispersion using an adsorption method. UV/ozone-treated sensors were first coated with poly-l-lysine (PLL) solution (0.1% w/v) via adsorption for 15 min followed by rinsing and nitrogen-drying. A single deposition of CLPs at 1.5 g/L concentration was applied onto PLL-coated sensors via adsorption for 30 min followed by rinsing and nitrogen-drying, resulting in CLP-coated sensors for QCM-D measurements. PLL-coated sensors were used as a control.

High-resolution images of the prepared thin films were obtained using a MultiMode 8 atomic force microscope (AFM) with a NanoScope V controller and an E scanner (Bruker, Billerica, MA). The images were acquired in air using tapping mode and NCHV-A probes (Bruker). NanoScope 8.15 or NanoScope Analysis 1.5 software (Bruker) were used for image analysis. The only image correction applied was flattening up to order 1.

### Adsorption of Keratin on Prepared Thin Films

Prior to the QCM-D measurements, a low-Mw keratin fraction was dissolved in several buffers: SPB (pH 7, 50 mM, 150 mM, 500 mM), SAB (pH 5, 50 mM), and McIlvaine buffer (pH 3, about 40 mM). Insoluble keratin was separated by centrifugation (3000 rpm for 5 min), and the solubility was measured as described earlier to ensure that all samples had the same concentration (0.1 mg/mL) in the adsorption studies.

Adsorption of keratin on the prepared lignocellulosic thin films was studied using a QCM-D E4 (Q-Sense, Sweden) in continuous flow mode at room temperature. The sensors with the deposited films were placed in the QCM-D chambers and, after determining their resonance frequencies and overtones, the films were allowed to equilibrate in the corresponding buffer for 1–2 h, injected at 0.1 mL/min flow rate, until a stable baseline was obtained. Upon the stabilization process, all the thin films showed slight increase in Δ*f* values (between 4 and 6 Hz) and decrease in Δ*D* values (between 0.5 and −0.3). After the baseline stabilization, the keratin solution was pumped with a 0.1 mL/min flow rate for 60 min. This was followed by a rinsing step with buffer for approximately 60 min, where reversibly adsorbed keratin was removed. Two or three parallel measurements were carried out for each sample. QCM-D allows the simultaneous measurement of the frequency shift and dissipation factor during the keratin adsorption. The mass adsorbed on the sensors causes a decrease in the resonant frequency. The sensed mass (Δ*m*) is proportional to the frequency change (Δ*f*) of the oscillating sensor if the mass is small compared to the mass of the sensor, does not slip on the electrode, and is sufficiently rigid and/or thin to have negligible internal friction. In this case, Sauerbrey relation^31,32^ can be used to calculate the sensed mass:
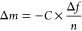
4where Δ*f* is the change in the frequency (Hz), Δ*m* is the change in the mass (mg × m^–2^), *C* is the mass sensitivity constant (*C* = 0.177 mg × m^–2^ × Hz^–1^ at 5 MHz), and *n* is the overtone number (*n* = 1,3, ...).

The increase in mass leads to a damping of the oscillation and energy losses. The dissipation factor *D* can thus be defined as^33^
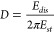
5where *E_dis_* is the dissipated energy and *E_st_* is the stored energy during one oscillation cycle. The dissipation change is given by Δ*D* = *D* – *D*_0_, where *D*_0_ is the dissipation of the sensor in the buffer before the measurement and *D* is the dissipation at any given time during the measurement.

## Results and Discussion

### Keratin Fractionation and Characterization

To perform adsorption studies, the feather keratin needs to be converted into a form that is soluble in an aqueous solution, and its solubility as well as structure should be understood to explain its adsorption behavior. Keratin has poor solubility due to disulfide cross–linking between and within polypeptide chains^8^ and tight packaging of the highly ordered secondary structures β-sheets and α-helices.^12^ The DES process was chosen to dissolve feather keratin in this study. We have previously shown that an aqueous DES composed of sodium acetate (NaOAc) and urea is able to dissolve feathers by disrupting the interactions within the keratin protein, cleaving disulfide bonds and partly degrading the polypeptide backbone of feather keratin.^10^ The degradation of the peptide backbones is not specific, and the used DES process yielded two keratin fractions with different molecular weight distributions.^10,11^ The yield of the keratin fraction with a higher molecular weight distribution was around 60%, while the yield of the keratin fraction with a lower molecular weight distribution and which was soluble in water together with diluted DES components was around 40%. After purification from the DES components and freeze-drying, the part of the low Mw keratin fraction that was further soluble in the aqueous buffers was used in the adsorption studies (Figure 1).

The weight average molar mass of the low Mw keratin fraction was 4500 ± 70 Da with a polydispersity of 1.7 (Figure S1, Supporting Information) when analyzed by SEC. The molecular weight of feather keratin is usually referred to be 10,000, Da which originates from the study of Woodin^34^ who extracted feather keratin with urea, phosphate, and reducing agent. This indicates that the DES treatment used here degraded the keratin more than the urea, phosphate, and reducing agent treatment. Previously, the Mw of high- and low-Mw keratin fractions were analyzed with matrix-assisted laser desorption ionization time-of-flight mass spectrometry (MALDI-TOF) revealing that the DES-treated keratin consisted of many different-sized keratin fragments.^11^ Similar observations of keratin molecular weight distributions have also been reported after *N*-methylmorpholine *N*-oxide (NMMO) treatment for feathers.^13^ Although the low Mw fraction was initially water soluble in the presence of diluted DES components, the low-Mw keratin fraction was not completely soluble in pure water or in the used buffers after dialyzing and freeze-drying. This indicated that further structural modification might have occurred during the drying process or the absence of the diluted DES components decreased the solubility. The weight average molar mass was measured from keratin, which was soluble in SPB buffer (pH 7, 150 mM), and it was 1200 ± 300 Da (Figure S2, Supporting Information), indicating that only the fraction with a very low Mw was soluble after drying. The average size of amino acids is 110 Da, and by dividing the measured weight average molar mass of keratin with the size of an amino acid (1200/110 Da = 11), it can be concluded that the average soluble keratin is approximately 11 amino acids in length. Hence, the keratin fraction studied was rather a peptide than a protein, and henceforth it is called keratin peptides in this work.

**Figure 2:**
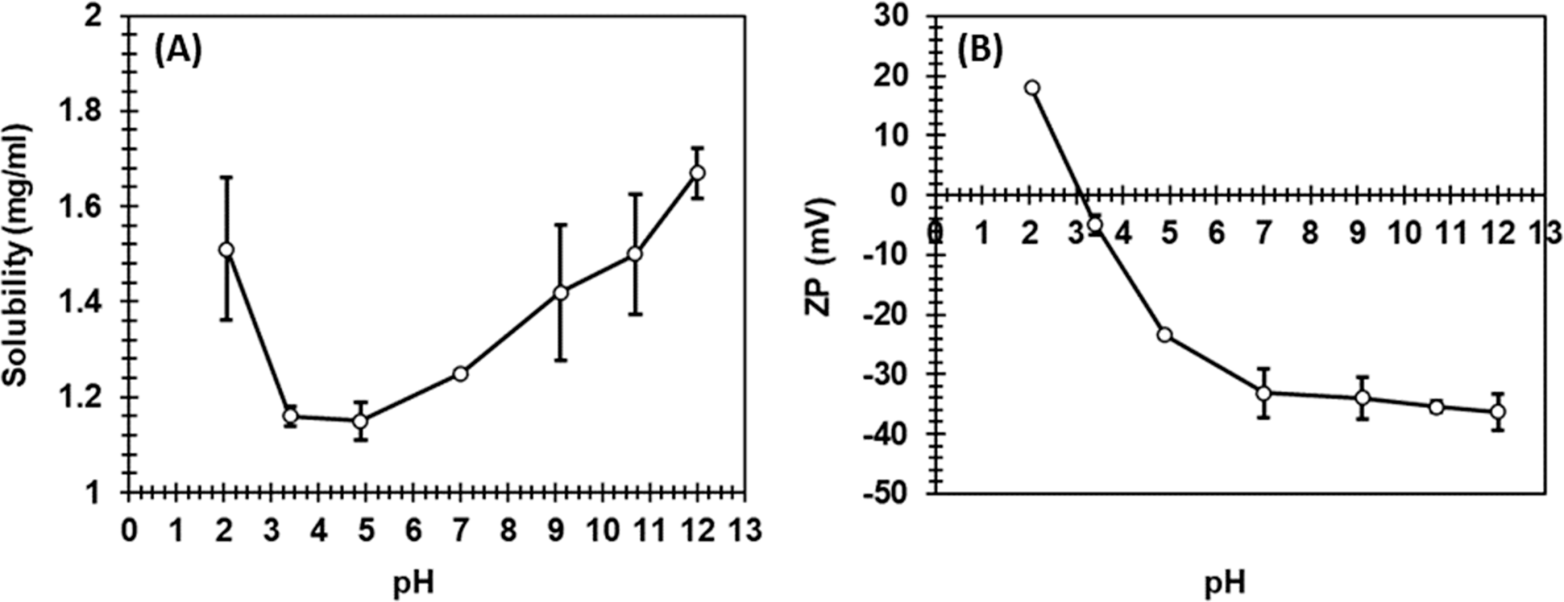
(A) Solubility and (B) zeta potential of the keratin peptides as a function of pH. For the experiments, 2 mg keratin was added to 1 mL of water with pH adjusted using HCl or NaOH solutions.

The solution pH and ionic strength also affected the solubility and the net surface charge of proteins; thus, the solubility and zeta potential of the keratin peptides were measured as a function of pH (Figure 2), and the solubility was also measured in different buffers (Table 1). The solubility of keratin peptides was the highest at pH 12 and started to decrease at lower pH values until it started to increase again at pH 2. The lowest solubility was obtained at pH values between 3 and 5. The zeta potential can be used to estimate the net surface charge of the particles. From Figure 2, it can be seen that at pH 2, the zeta potential of keratin was positive, having a value of +18 mV, and when the pH increased to pH 12, the zeta potential decreased reaching a value of −36 mV. Solubility and zeta potential values were well in line demonstrating that keratin peptides carrying a charge had a higher solubility. The zeta potential of keratin peptides was also measured in 200 mM SPB (pH 7) and SAB (pH 5) giving values of −8 and −6 mV, respectively. The solubility in different buffers varied from 0.37 to 1.35 mg/mL (Table 1). The highest solubility of the tested buffers was 1.35 mg/mL in 50 mM SPB, pH 7, and the lowest solubility is 0.37 mg/mL in a pH 3 McIlvaine buffer. As expected, increasing the salt concentration from 50 to 500 mM lowered the keratin peptide solubility. At a pH close to the isoelectric point (IEP) of a protein, when the net charge of the protein is near zero, or at high salt concentrations where electrostatic double layer repulsion between charged molecules is screened, the protein may aggregate due to lack of long-range repulsion between charged residues, leading to poor solubility.^35^ Around pH 3, the net charge was zero, indicating the IEP of the keratin peptide. Similar IEPs have been previously reported for keratin.^36,37^ Sharma et al.^36^ prepared two types of keratin microparticles using an isoelectric precipitation at pH 3.5 and 5.5, while Zhang et al.^37^ collected precipitated acid hydrolyzed keratin at pH 3.22 and pH 5.55, indicating that the processed keratin stream consisted of different types of keratin fragments, which precipitated at different IEPs.

**Table 1:** Solubility of the Keratin Peptides in the Buffers Used in QCM-D Studies

Buffer	pH	Ionic strength (mM)	Solubility (mg/mL)
SPB	7	50	1.3
SPB	7	150	1.0
SPB	7	500	0.4
SAB	5	50	0.7
McIlvaine buffer	3	40	0.4

**Figure 3:**
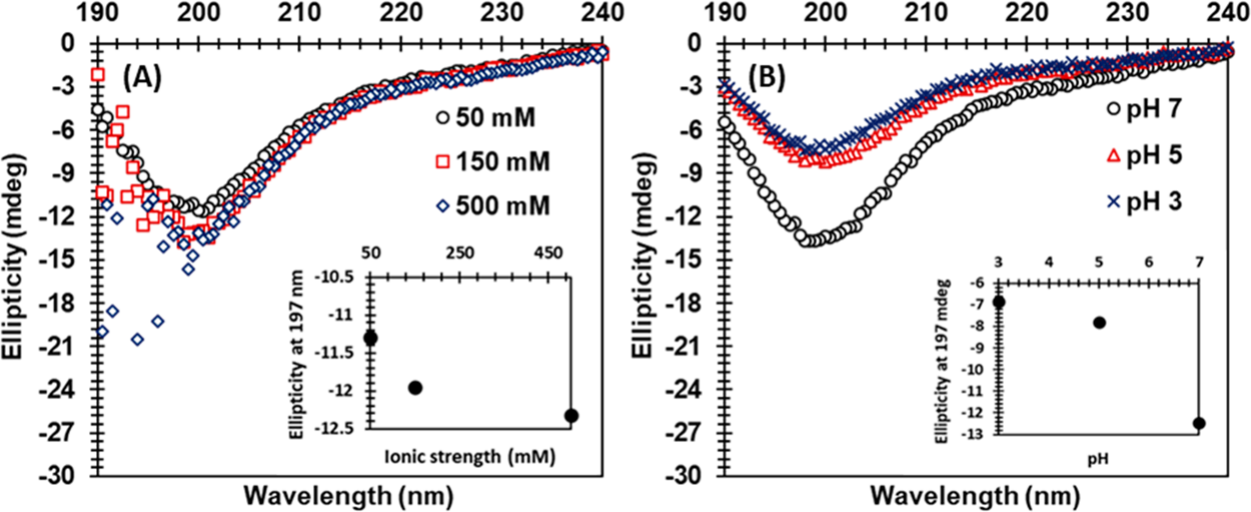
CD spectra of keratin peptides in (A) 50, 150, and 500 mM SPB (pH 7) and (B) at pH 7 (SPB, 50 mM), pH 5 (SAB, 50 mM), and pH 3 (McIlvaine buffer, 40 mM). The inset in (A) is the ellipticity at 197 nm vs ionic strength, and the inset in (B) is the ellipticity at 197 nm vs pH.

CD was used to analyze the secondary structure of the keratin peptides as a function of ionic strength and pH (Figure 3). The strong negative bands at 200 nm in all CD spectra indicate that the keratin peptides had a random coil and not a folded structure. This is well in line with previous findings that showed that feather keratin solubilized with urea and bisulfite also had a random coil conformation and showed similar CD spectra.^38^ The slight changes in the CD spectra as a function of ionic strength and pH were most probably related to the change in net charge of the peptides and solubility, and not due to actual conformational changes in the polypeptide backbone. Feather keratin is a fibrous structural protein whose polypeptide chains are tightly packed as α-helix and β-sheet structures.^39^ Keratin has a large number of cysteine residues, which can form strong covalent disulfide bonds within a keratin molecule as well as with other keratin molecules leading to inter and intra cross-linking and making keratin rigid.^40^ Besides the decrease in molecular weight, a part of this ordered structure and cysteine residues of keratin were lost during the processing.^10,12,13^ This could indicate that the keratin used in this study transformed from a highly ordered protein to more labile, unstructured peptides.

Amino acid content determines the conformation and stability of proteins and thus plays an important role in the adsorption behavior.^41^ The keratin solution used in this study contained differently sized keratin fragments, which made it difficult to determine the exact amino acid composition and order. However, it is known that water-soluble keratin peptides are rich in negatively charged glutamic and aspartic acids, as well as hydrophilic serine and hydrophobic proline. However, amino acids such as positively charged arginine and hydrophobic glycine and leucine have also been observed to a significant extent in the structure.^13^ It must be noted that although at pH 7, keratin peptide (Figure 2) has a negative net surface charge, some amino acids such as arginine and glutamine carry a positive charge. Thus, the amino acid composition suggests that water-soluble keratin peptides are amphoteric. They are also suggested to be amphiphilic and surface active due to presense of both polar and nonpolar structures.^42^ The CD spectra showed that keratin peptides had a random conformation making them labile, which enhances adsorption. Labile proteins are susceptible for protein unfolding and able to adopt a conformation, which is favorable for adsorption, whereas movement of rigid proteins is more restricted.^41^

### Adsorption on Cellulose and Lignin

The adsorption behavior of keratin peptides was studied on thin model films prepared from regenerated cellulose, dissolved (diss.) lignin, and colloidal lignin particles (CLPs) using the QCM-D technique. The preparation methods of thin films were selected to correspond as closely as possible to the pure substances and to ensure their suitability for QCM-D studies.^28−30,43^ Moreover, polystyrene (PS) and poly-l-lysine (PLL) thin films were used as controls since they were used as anchor layers.

Diss. lignin and CLP exhibited high keratin adsorption with Δ*f*_3_ values of −56 ± 2 Hz and −64 ± 1 Hz at the plateau region at pH 7 and 150 mM, respectively (Figure S3 in the Supporting Information). The majority of keratin was adsorbed within the first 15 min. On the contrary, keratin had low adsorption to cellulose, exhibiting a Δ*f*_3_ value of only −9 ± 1 Hz at the plateau region (Figure S3 in the Supporting Information). Keratin also adsorbed onto both of the controls, PLL and PS, with Δ*f*_3_ values of −38 and −34 Hz, respectively. However, the adsorption was clearly different from the adsorption onto lignin and cellulose thin films, indicating that the thin film preparation was successful, and keratin was interacting with cellulose or lignin and not only with the underlying layer. This was further supported by the AFM images of the prepared films that exhibited uniform layers (Figure S4 in the Supporting Information). AFM images of the prepared thin films were also in line with previously reported AFM images of cellulose,^28,43^ lignin,^30^ and CLP^29^ thin films.

**Figure 4:**
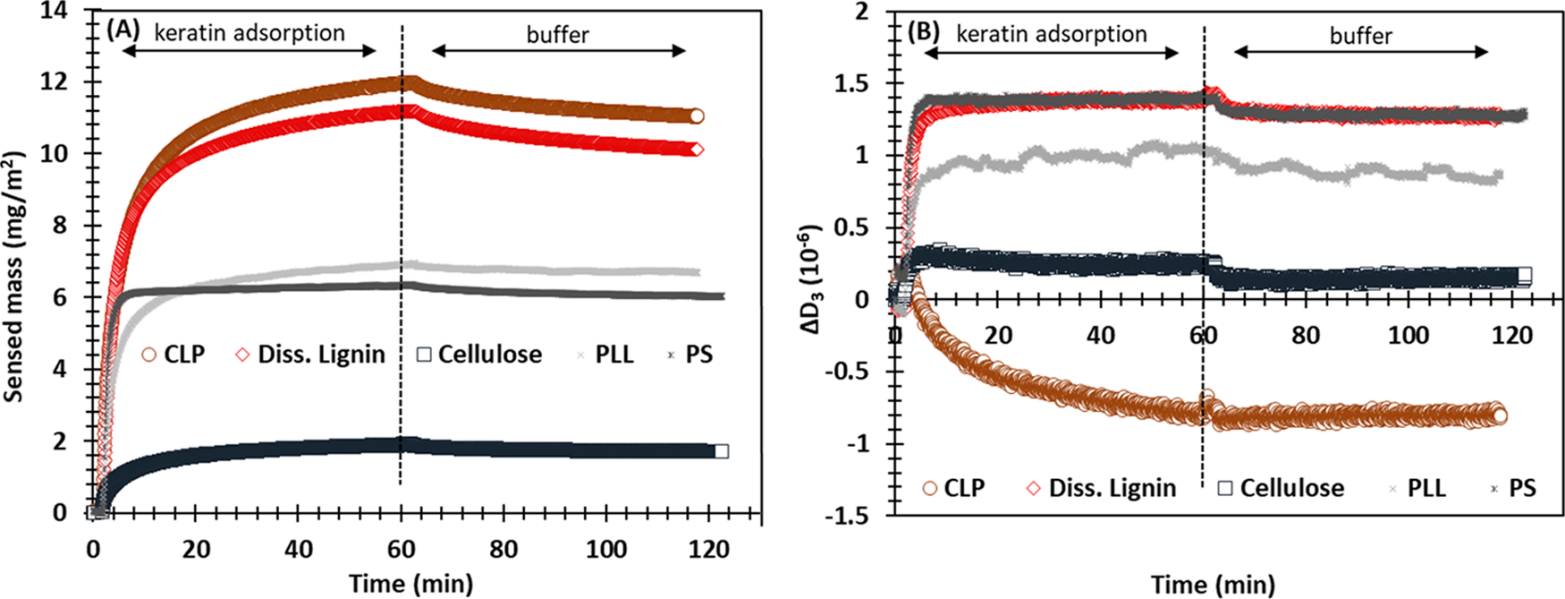
QCM-D detection of the adsorption of keratin peptides onto PS (control), PLL (control), CLP, diss. lignin, and cellulose thin films: (A) sensed masses calculated using eq 4 and (B) dissipation changes vs time at the third overtone at pH 7 and 150 mM.

Using the Sauerbrey eq 4 Δ*f_3_* values could be converted into sensed mass. The sensed adsorbed mass of keratin was 10.0 ± 2.0 mg/m^2^ onto diss. lignin, 11.2 ± 0.1 mg/m^2^ onto CLP, and 1.7 ± 0.1 mg/m^2^ onto cellulose at the plateau region (Figure 4). The Sauerbrey equation applies if the adsorbed layer is rigid. Usually, when the dissipation changes are low, a film can be considered rigid. However, another way to find out whether the film can be treated as rigid is to study the resonant frequency and the harmonic numbers.^44^ If the frequency changes are overtone dependent, the films should be treated with a viscoelastic model.^44^Figure S5 (Supporting Information) shows that the frequency changes are overtone independent (overtones 3, 5, and 7 are shown). Due to the above mentioned reasons, the adsorbed layers were considered to be dominantly rigid rather than viscoelastic and hence the Saurbrey equation was applied to calculate the sensed mass. Due to their small size, the adsorbed keratin peptides were not visible on the surfaces when imaged with AFM (Figure S4 in the Supporting Information) but the root-mean-square roughness (*Rq*) changed from 4.21 to 6.18 nm.

The change in dissipation, Δ*D_3_*, at the plateau region after the keratin adsorption onto diss. lignin and cellulose were (1.4 ± 0.2) × 10^–6^ and (0.2 ± 0.1) × 10^–6^, respectively. Δ*D_3_* can be used to evaluate the softness of the adsorbed layer. The very low values observed for Δ*D*_3_ suggest that the small keratin peptides adsorbed rigidly on the surfaces, forming a dense layer. The very low change in dissipation observed for cellulose is connected to the low adsorption of keratin peptides on that model film. In other words, when the adsorption is low, the keratin peptides are expected to adsorb in a flatter conformation because they do not need to compete between each other for the large surface area available, and consequently, the increase in the dissipation factor is minimal. The Δ*D_3_* value for CLPs at the plateau region was negative (−1.0 ± 0.3) × 10^–6^ indicating that the film on the sensor became denser although the mass on the CLP film increased after the keratin adsorption. A similar phenomenon has been observed before, and the decrease in dissipation is most probably related to the release of bound water from the CLPs upon adsorption of keratin.^29,45−47^ However, the shape of the CLPs may be partly responsible for such a release as such a phenomenon did not take place on the substrate made of dissolved lignin. This has also been observed previously when the adsorption of cationic lignin was studied on dissolved lignin and CLP substrates.^29^

From the AFM images (Figure S4 in the Supporting Information), it could be seen that when the image projected surface area was 25 μm^2^, the real surface areas were 25.2, 36.7, and 25.1 μm^2^ for lignin, CLPs, and cellulose, respectively. Using these values and assuming that keratin peptides are 11 amino acids long with one amino acid having a length of 4 Å, shape of a circle, and molecular weight of 110 Da, we were able to calculate the theoretical surface coverage when keratin peptides adsorb in flat conformation. These values were 1.47, 2.14, and 1.46 mg/m^2^ for lignin, CLPs, and cellulose, respectively. Considering the sensed adsorbed masses of keratin peptides (10.0 ± 2.0 mg/m^2^ onto diss. lignin, 11.2 ± 0.1 mg/m^2^ onto CLP, and 1.7 ± 0.1 mg/m^2^ onto cellulose) and the low dissipation values, it appears that the substrates were fully covered by the keratin peptides and on lignin substrates, they adsorbed on top of each other. However, these calculations neglect possible effect of bound water and include some assumptions, hence more studies are needed before drawing any firm conclusions.

Peptide adsorption on a solid surface is a complex phenomenon affected both by the change in entropy and enthalpy. The entropy gain upon adsorption to the solid substrate is mainly due to released solvent molecules from the surface (dehydration) or changes in the protein structure leading to increased conformational entropy.^48^ In the case of charged molecules and surfaces, there is also a release of counter ions increasing the net entropy. Hence, the entropy gain is one important driving force for adsorption. The enthalpic interactions are more complex, but hydrophobic interactions as well as electrostatic interactions are known to have an important role in protein adsorption.^41,49^

As in this study with keratin peptides, higher non-specific adsorption of the two main proteins in soy on lignin compared to cellulose have also been previously noticed.^50^ These soy proteins are also amphiphilic and include cationic amino acids, but compared to keratin peptides, they have a higher molecular weight (about 180 and 320–350 kDa) and a different secondary structure, which affect the adsorption behavior.^50^ The sensed masses of keratin peptides were 10.0 ± 2.0 mg/m^2^ for adsorption on diss. lignin, 11.2 ± 0.1 mg/m^2^ for CLP, and 1.7 ± 0.1 mg/m^2^ for cellulose. Previously, using similar conditions, soy protein adsorption on diss. lignin surfaces had been reported to be 15.6 and 20.5 mg/m^2^,^50^ while gelatin, casein, BSA, conalbumin, and albumin adsorption on lignin have been reported to be 8.2, 7.7, 6.7, 3.3, and 1.6 mg/m^2^,^51^ respectively. Protein adsorption on the lignin surface is not yet understood, but electrostatic and hydrophobic interactions, certain specific amino acids, and the conformation of the protein structure are all speculated to have a decisive role.^52,53^

At pH 7, keratin peptides, as well as cellulose^54^ and lignin surfaces,^55^ have a negative net surface potential. Hence, in principle the observed adsorption could not be explained by electrostatic attraction between the substrate and peptide of the opposite charge. Nevertheless, the labile structure of keratin peptide (Figure 3) may enable the attraction between remaining positively charged amino acids and the negatively charged substrate. If this is case, the low adsorption on the cellulose may indicate that the thin cellulose films are weakly negatively charged. Indeed, it has been reported that cellulose has a low negative charge^54^ compared to lignin.^55^ Surface potentials as low as −15 and −2 mV have been reported for cellulose model films (similar to the ones used in this work) interacting between each other or with a mica substrate, respectively, in 0.1 mM KBr.^56^ In contrast, the zeta potential of CLPs has been reported to be approximately −40 mV at pH 7 and negative at pH above 2.^55^

The water contact angle on regenerated cellulose has been reported to be 31 ± 3°.^54^ This indicates a hydrophilic surface, which could explain the absence of strong hydrophobic interactions and the poor adsorption of keratin on cellulose. The water contact angle on lignin thin film has been reported to be around 60°,^29^ supporting the possibility for some hydrophobic attraction between the keratin and lignin. However, the water contact angle on the CLP surface is only 17 ± 1°^29^ and yet a slightly higher adsorption of keratin was observed on the CLP substrate compared to the lignin substrate and much higher compared to that on the cellulose substrate. Partly, this can be explained by the higher accessible surface area on a substrate constructed by spheres, compared to a thin flat film, or higher dehydration as could be seen from Δ*D_3_* values, but another possible reason could be more accessible carboxyl and hydroxyl groups at the CLP surface. It is also suggested that cellulose thin films are in an amorphous state, which means that its hydroxyl groups are available,^28,57^ thus indicating that hydroxyl groups were not responsible for adsorption.

Yamaguchi et al.^58^ studied peptide adsorption on wood lignin surfaces and identified peptide sequences that have high affinity to lignin. Especially, peptides, which contained positively charged histidine, hydrophobic phenylalanine, non-polar proline, and polar serine residues, had high affinity to the lignin surfaces, and a highly flexible random coil structure allowed the key residues to be appropriately arranged in relation to the binding site in lignin.^58^ Especially, the interactions between the aromatic moieties of lignin and proline rings of peptides^51^ as well as the interactions between positively charged amino acids and negatively charged lignin groups (i.e., carboxyl groups) could explain the adsorption. It could be assumed that the specific amino acid content as well as the random coil conformation of keratin peptides (Figure 3) favored adsorption to lignin.

Enzyme adsorption on cellulose is widely studied for carbohydrate active enzymes. Cellulases, an important component in lignocellulose degrading enzyme cocktails, can either cleave the amorphous or crystalline regions of cellulose. The adsorption of these enzymes onto the substrate surface can take place via a specific cellulose-binding domain (CBD). Typically CBDs, which recognize insoluble cellulose, contain a planar surface with three aromatic amino acids involved in cellulose binding.^59−61^ As the binding of keratin to cellulose after the DES treatment was low, it is unlikely that keratin peptides contained these specific amino acid conformations characteristic of CBDs. Bovine serum albumin (BSA) has been previously used to study the non-specific protein adsorption on cellulose surfaces with QCM-D and surface plasmon resonance (SPR). Near pH 7, the adsorption of BSA on cellulose was very low.^46,47,62^ Our results are in line with previous observations of poor interaction between cellulose and feather keratin when preparing cellulose–keratin filaments.^24^

**Figure 5:**
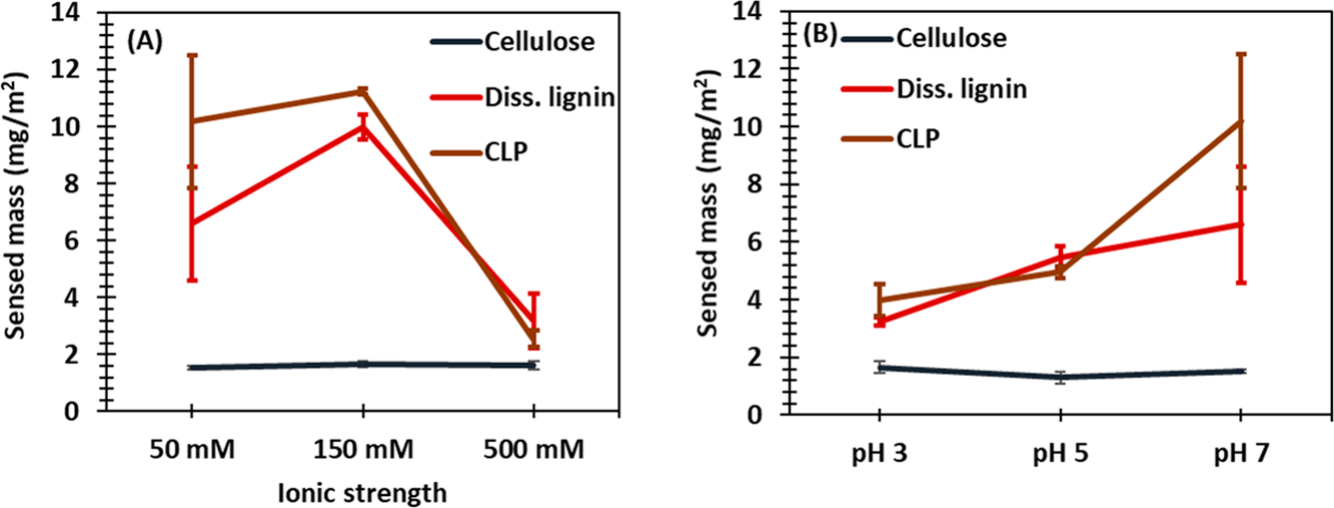
Adsorption of keratin peptides calculated from the Sauerbrey equation onto CLPs, diss. lignin, and cellulose thin films at different (A) ionic strengths (pH 7) and (B) pH values (ionic strength 40–50 mM).

To gain further understanding, the effect of different adsorption environments was studied. Conditions were varied using different buffers with different pH values and ionic strengths to alter the physicochemical environment in a controlled manner. Keratin peptides were dissolved in different buffers with pH 3–7 and 50–500 mM electrolyte concentrations. The keratin solubility was different in the different buffers mostly due to the pH-dependent charge of the keratin. Thus, the solubility of the keratin in the different environments was measured after the dissolution and centrifugation and the highest solubility was in SPB (pH 7, 50 mM) (Table 1). All keratin solutions were diluted to 0.1 mg/mL for QCM-D experiments. Figure 5 represents the sensed masses calculated from Δ*f*_3_ values obtained using the Sauerbrey eq 4.

Figure 5 shows that an increase in ionic strength from 50 to 500 mM or a change in pH from 7 to 3 did not affect the adsorption of keratin peptides on the cellulose substrate. It was low regardless of the pH or ionic strength of the media. Previously, non-specific protein adsorption on cellulose surfaces has been increased by ensuring the electrostatic attraction by opposite charges.^47,62^ However, with the IEP of the studied keratin fraction being as low as pH 3, it was not practical to obtain opposite charges for the keratin peptides and cellulose surface by only changing the pH. It has been reported that the adsorption of BSA on cellulose is the highest at its IEP,^47,62^ but it seems that keratin adsorption on cellulose was not favorable even then.

In contrast, adsorption of keratin peptides on the lignin surfaces exhibited remarkable differences with respect to change in the ionic strength and pH (Figure 5). When the ionic strength was increased from 50 to 500 mM, the sensed mass decreased from 6.6 ± 2.0 to 3.2 ± 1.0 mg/m^2^ on diss. lignin and from 10.2 ± 2.3 to 2.6 ± 0.3 mg/m^2^ on CLP film. When pH was increased from 3 to 7, the sensed mass increased gradually from 3.2 ± 0.1 to 6.6 ± 2.0 mg/m^2^ for diss. lignin and from 4.0 ± 0.6 to 10.2 ± 2.3 mg/m^2^ for CLP. Due to the expected long-range electrostatic repulsion at higher pH values and lower ionic strengths,^47^ the increase in protein adsorption was not fully expected. It was anticipated that at pH 3 or at high ionic strengths, the adsorption of keratin peptides would have been higher than at higher pH values or low ionic strengths because the net surface charge of keratin was zero and the electrostatic interactions were screened allowing a larger amount of protein to accommodate at the surface.^49^

One explanation for this unexpected behavior could still be favorable electrostatic interaction since it should be noted that although the net charge of the keratin peptides was negative, there were some amino acids with positive charge that could favor the adsorption. As the pH increases from 3 to 7, the carboxylic groups on lignin are deprotonated and could interact with the cationic amino acids. As the salt concentration increases from 50 to 500 mM, the ions screen the attraction between positive and negative groups. Interestingly, there seems to be a maximum at 150 mM. Increasing the ionic strength to some extent may screen the long-range electrostatic repulsion between negatively charged keratin peptides, allowing keratin peptides to adsorb on the lignin substrate closer to each other, which turns into higher adsorption at 150 mM, while a further increase in electrolyte concentration also screens the favorable interactions, decreasing the adsorption.

Another possible reason for the observations may be the varying molecular weight. The solubility of processed feather keratin is related to the molecular weight, and high molecular weight keratin fragments have lower solubility.^10,11,13^ Hence, we may assume that if the solubility is poorer, like in aqueous media of low pH or high salt (Table 1) when the net charge of keratin is negligible, the soluble keratin will consist of mainly keratin peptides with very low molecular weight (Figures S1 and S2, Supporting Information) with random coil conformation (Figure 3). This could be one reason for the lower sensed mass. It could then be speculated that the structural properties, as well as non-charge meditated interactions, have a high impact on keratin adsorption on lignin surfaces. This has been also suggested in other studies in which non-specific protein adsorption is studied on lignin surfaces.^50,51^

Especially, labile proteins may adsorb onto surfaces even when electrostatic repulsion is present, and in this case, the adsorption is probably related to conformational rearrangements leading to an entropy gain.^63^ Malmsten^35^ found that the polymer adsorption increased and become more favorable when the molecular weight of the polymer increased. This was explained by decreased entropy loss upon polymer adsorption with increased molecular weight. In simpler systems, peptides have been found to be in line with this effect, while in more complex systems such as polyelectrolytes and proteins, this molecular weight effect is less distinct.^35^ The increased adsorption at higher molecular weight could also be related to the higher amount of available sites in keratin for adsorption. It is speculated that molecules with higher molecular weight or more extended conformation may have higher adsorption onto the surface due to the higher number of available sites^64^ including hydrophobic and positively charged groups. At IEP or high ionic strengths, the keratin solution most probably contained keratin peptides with lower molecular weight and when the negative net charge increased, it allowed solubilization of keratin peptides with higher molecular weight.

**Figure 6:**
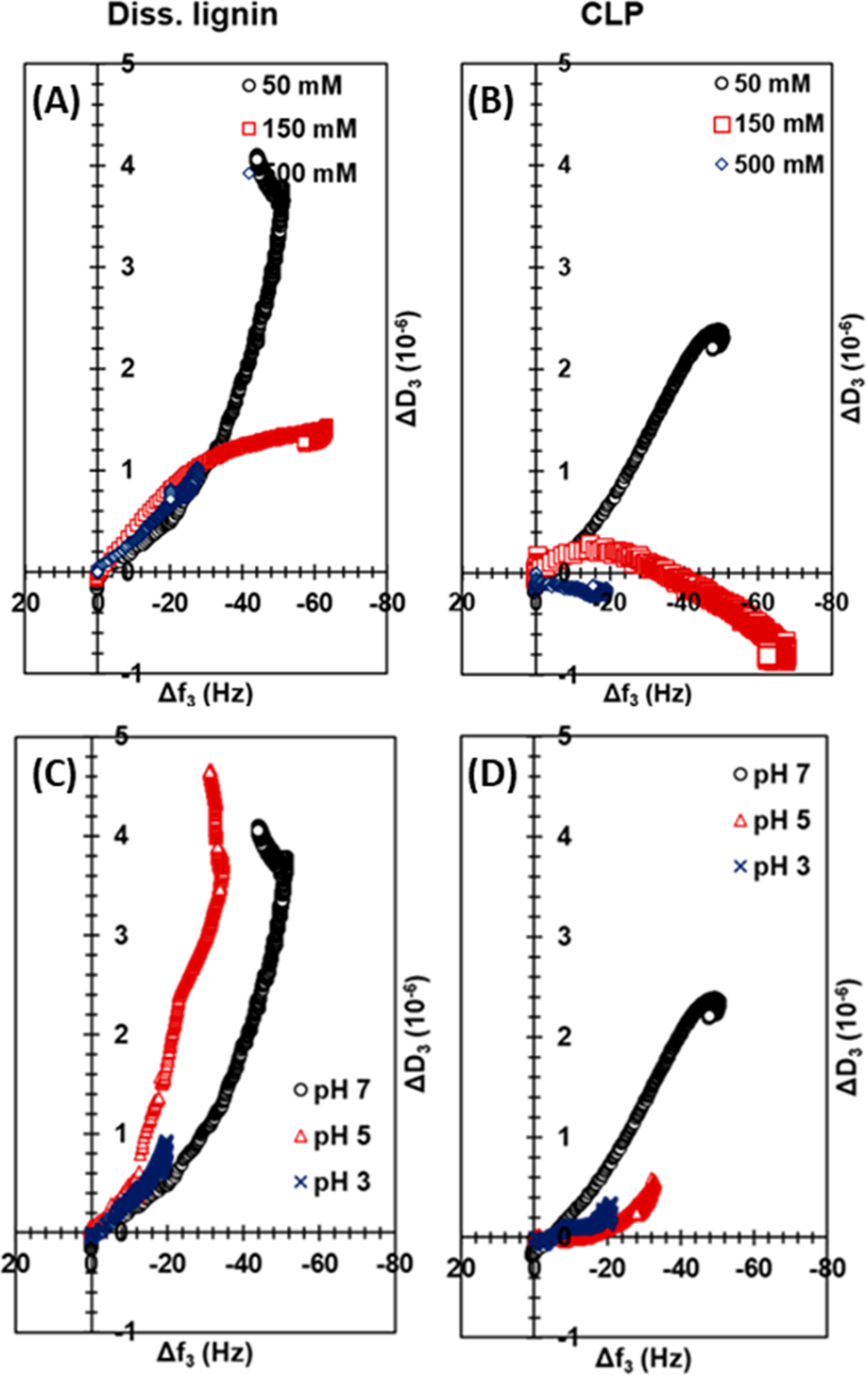
Change in dissipation factor as a function of the change in frequency for keratin peptides when adsorbed onto (A, C) dissolved lignin and (B, D) CLP-coated gold sensors at different (A, B) ionic strengths and pH 7 and (C, D) different pH values at 150 mM.

A systematic alteration in the pH and ionic strength caused a corresponding change in the frequency and dissipation upon the adsorption. To further understand the viscoelastic properties and possible changes in the layer properties during the adsorption process, Figure 6 shows the change in dissipation (Δ*D*) as a function of the shift in frequency (Δ*f*). At the lowest ionic strength of 50 mM, when both keratin peptides and surfaces were negatively charged, the dissipation changes were the largest, indicating that the adsorbed keratin layers on both diss. lignin (Δ*D* = 4.0 × 10^–6^) and CLP films (Δ*D* = 2.3 × 10^–6^) were the most hydrated and swollen. Charged molecules carry more bound water with them compared to uncharged molecules due to fact that the electrostatic repulsion proteins also adsorb in a more extended conformation.^65^ At the high salt concentration (500 mM), the electrostatic interactions were screened and at IEP (pH 3), keratin was uncharged leading to a less hydrated layer. Interestingly, for CLPs, at 50 mM SPB (pH 7), the layer seems to become more swollen when more keratin is adsorbed, while at 150 mM, there is a clear decrease in the Δ*D*/Δ*f* slope at around −30 Hz suggesting a densification of the layer. As discussed earlier, this could be related to the removal of bound water from the CLP surface upon the adsorption. It can be concluded that the adsorbed layers of keratin on diss. lignin were softer compared to the adsorbed layers on CLPs at the same conditions as depicted by the dissipation values, even though there was not much difference in the adsorbed amounts. As expected, Δ*D*/Δ*f* profiles for cellulose did not show any significant difference due to the low adsorption of keratin peptides (Figure S6 in the Supporting Information).

**Figure 7:**
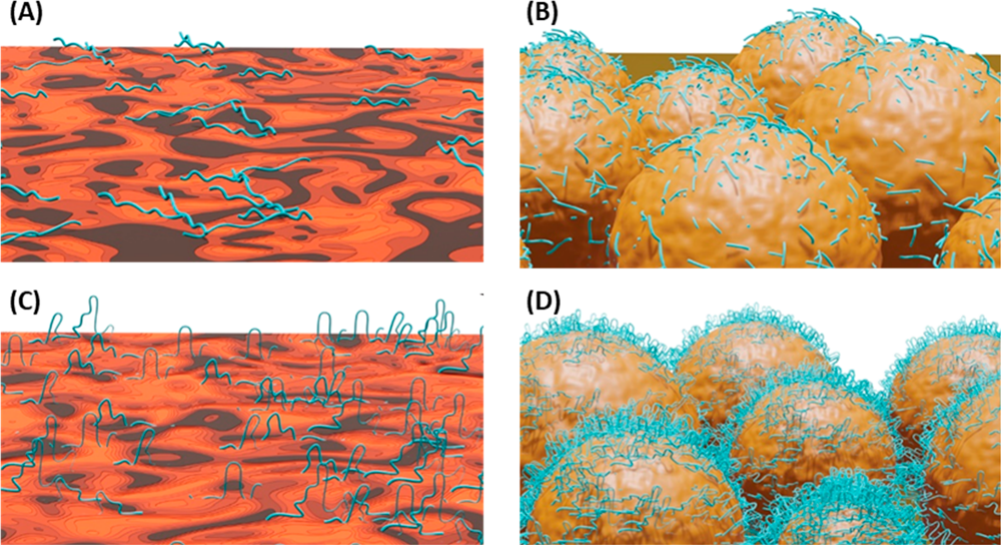
Illustrative graphics summarizing the main observations regarding the adsorption of keratin peptides on (A, C) diss. lignin and (B, D) CLP thin films. In (A, B), the electrostatic interactions were screened at high ionic strength or when the net charge of keratin is negligible, leading to low adsorption of small keratin peptides. In (C, D), both keratin and lignin are negatively charged and the ionic strength is low, resulting in solubility of bigger keratin peptides and in larger adsorption in a more extended conformation than in (A, B).

By studying the interactions between keratin peptides and surfaces made of lignocellulosic building blocks using the surface sensitive QCM-D technique, we were able to shed some light on the main driving force for keratin adsorption (electrostatic interactions and entropy gain) and understand the main factors affecting their interactions. Figure 7 visualizes our hypothesis of how the DES treated feather keratin adsorbs on lignin surfaces and how the surface morphology, molecular weight of the keratin, and nature of the media affects the mass and conformation of the adsorbed layer. In the absence of electrostatic interactions (screened by high ionic strength or pH close to the IEP of keratin), the adsorption of keratin peptides is low, and the adsorbed layers are not very hydrated and extended. In contrast, when the electrostatic interactions are present (*i.e.*, both keratin and lignin are negatively charged and the ionic strength is not very high) more keratin is adsorbed forming more hydrated and extended layers. The higher adsorption in this situation can be both electrostatically and entropically driven due to the existence of some cationic amino acids in the keratin peptides and the expected presence of larger peptide molecules in the medium, respectively. This fundamental understanding could ultimately lead to better product design for applications where structural proteins play an important role. Keratin could bring additional properties to lignocellulosic products such as wound dressings, scaffolds, drug carriers, textiles, hydrogels, electronic materials, and absorbents.

## Conclusions

Proteins fractionated from industrial side-streams shows potential to enhance the functionality of bio-based lignocellulosics increasing their applicability, especially in the biomedical field. However, a fundamental understanding of their interactions is essential to pave the way for combining protein with lignocellulosic building blocks at the nanoscale design stage to enable good affinity between components and the best material performance. In this study, an environmentally friendly and scalable DES process was used to obtain keratin from feathers. The *in situ* adsorption and affinity of keratin peptides for cellulose, lignin, and colloidal lignin particle model surfaces was systematically analyzed by the surface-sensitive QCM-D technique. Media-dependent solubility of keratin peptides was observed, and the net surface charge was found to play a pivotal role in the solubility of keratin peptides. The solubility increased when keratin had a higher negative net charge, which also most likely allowed keratin peptides with a higher molecular weight to dissolve. The DES processed keratin peptides adopted a random coil conformation, which is considered as an advantage in adsorption. The interactions between cellulose and keratin peptides were found to be weak, and altering the physiochemical environment (pH, ionic strength) did not increase the adsorption. On the other hand, keratin peptides had a high adsorption on lignin surfaces, and the adsorption behavior could be modified by altering the physiochemical environment. The adsorption of keratin on lignin is a complex process, but it was anticipated that the structural properties including the amino acid content, the conformation, and the molecular weight of the keratin peptides played an important role in its adsorption. Especially, the properties of lignin-based materials could be improved with keratin due to the high keratin adsorption to lignin. Keratin combined with colloidal lignin particles is an interesting approach because the spherical morphology of the nanoparticles is beneficial in many practical applications. Keratin together with colloidal lignin particles could be used, *e.g.*, in the production of hydrogels for biomedicine and cosmetics, bio absorbent, or electronic materials, thus ensuring the value-addition for two industrial side-streams. On the other hand, the interactions between cellulose and keratin should be improved, *e.g.*, by surface modification or covalent cross-linking, to enable the use of keratin in cellulose-based applications.

## Supporting Information Available

SEC analysis of keratin, AFM images of thin films, overtone independence of frequency changes (QCM-D data), and Δ*D*/Δ*f* profiles for keratin adsorption on cellulose thin films (PDF)
