# Supplementary material for: Affinity of Keratin Peptides for Cellulose and Lignin: A Fundamental Study toward Advanced Bio-Based Materials
Source: Langmuir. 2022 Aug 5;38(32):9917–27. doi: 10.1021/acs.langmuir.2c01140 (PMC9387096; doi:10.1021/acs.langmuir.2c01140)
Supplement: Supplementary file 1 — la2c01140_si_001.pdf [file la2c01140_si_001.pdf]

Affinity of keratin peptides for cellulose and lignin: a fundamental study towards advanced bio-based materials

Emmi-Maria Nuutinen[†‡]*, Juan José Valle-Delgado*[‡]*, Miriam Kellock*[†]*, Muhammad Farooq*[‡]*, and Monika Österberg**[‡]

[†]Sustainable products and materials, VTT, Technical Research Centre of Finland, Tietotie 2, FI-02044, Espoo, Finland

[‡]School of Chemical Engineering, Department of Bioproducts and Biosystems, Aalto University, 02150, Espoo, Finland

*Corresponding author : monika.osterberg@aalto.fi, +358505497218

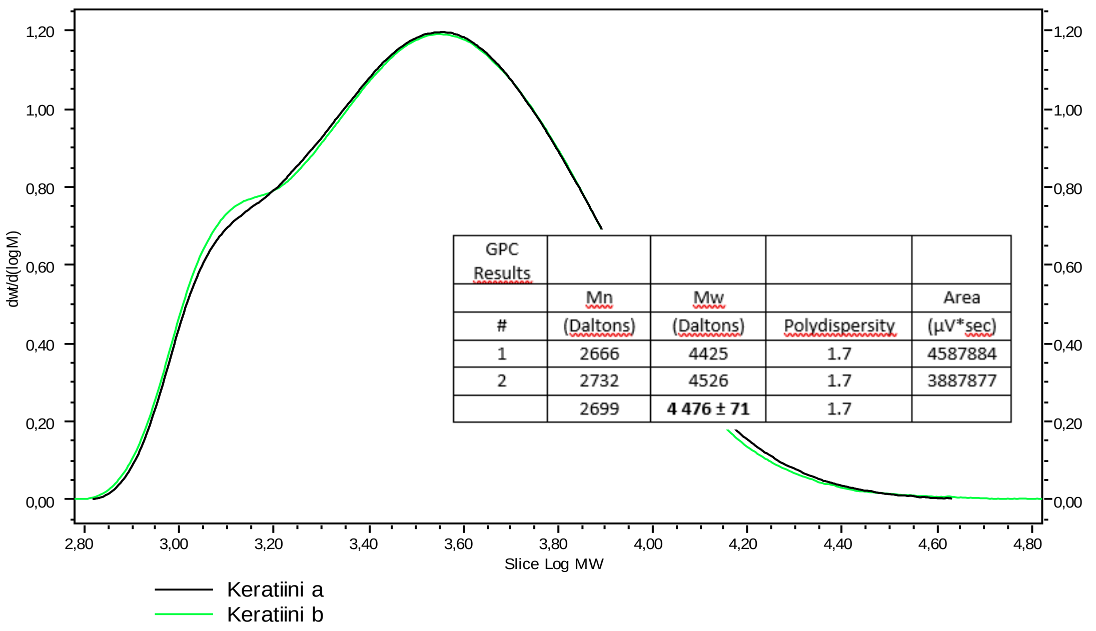

GPC Results				
	Mn	Mw		Area
#	(Daltons)	(Daltons)	Polydispersity	(µV*sec)
1	2666	4425	1.7	4587884
2	2732	4526	1.7	3887877
	2699	**4 476 ± 71**	1.7	

Figure S1. The molecular weight of the keratin fraction obtained from the DES fractionation.

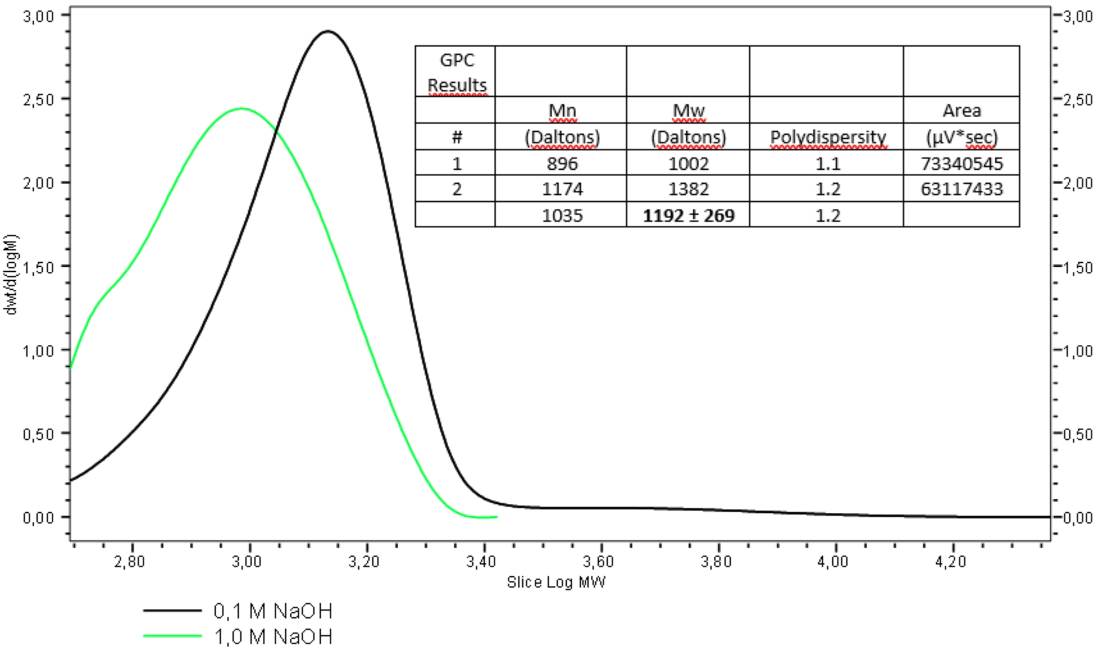

GPC Results				
	Mn	Mw		Area
#	(Daltons)	(Daltons)	Polydispersity	(µV*sec)
1	896	1002	1.1	73340545
2	1174	1382	1.2	63117433
	1035	**1192 ± 269**	1.2	

Figure S2. The molecular weight of the keratin fraction which was soluble in pH 7 150 mM sodium phosphate buffer and used in the QCM-D studies.

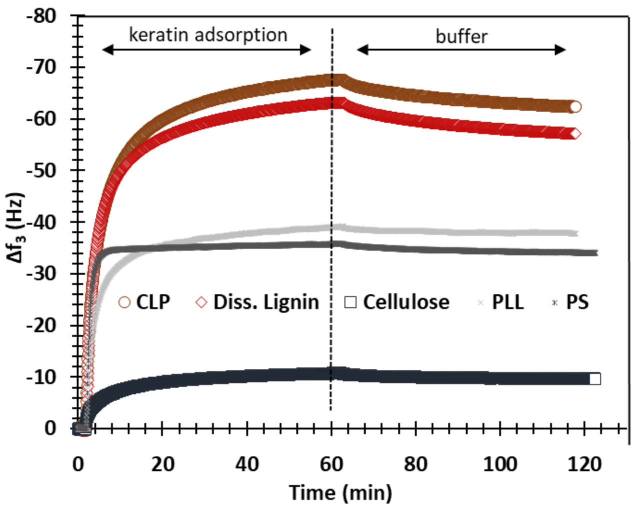

Figure S3. QCM-D detection of the adsorption of keratin peptides onto PS (control), PLL (control), CLP, lignin and cellulose thin films: frequency changes vs time at the third overtone at pH 7 and 150 mM.

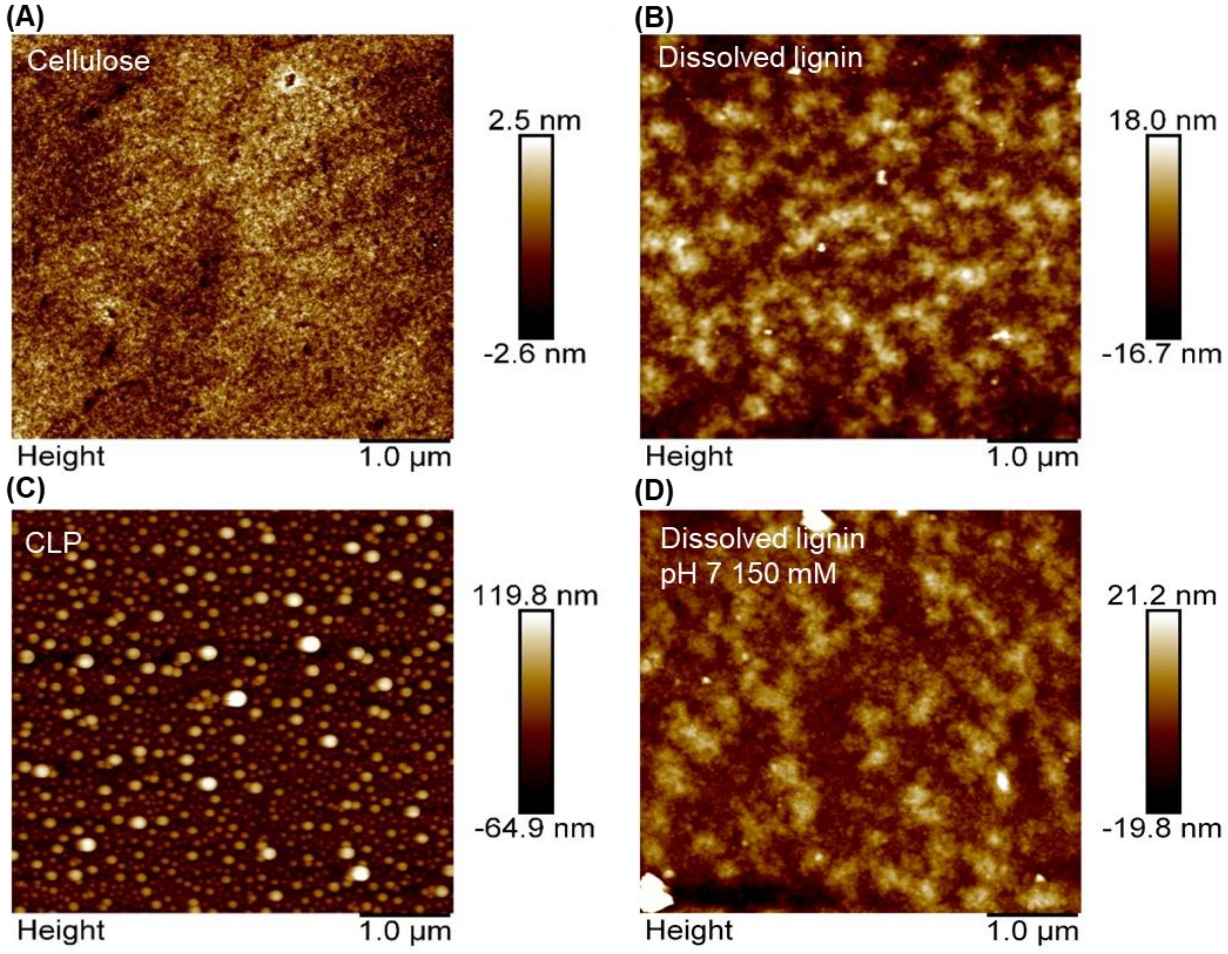

Figure S4. AFM height images for thin films of (A) cellulose, (B) lignin and (C) CLPs on PS (cellulose, lignin) or PLL coated QCM-D crystals measured in ambient air. (D) AFM height image measured in ambient air for the prepared substrate after keratin adsorption on soluble lignin substrate at pH 7 and 150 mM ionic strength.

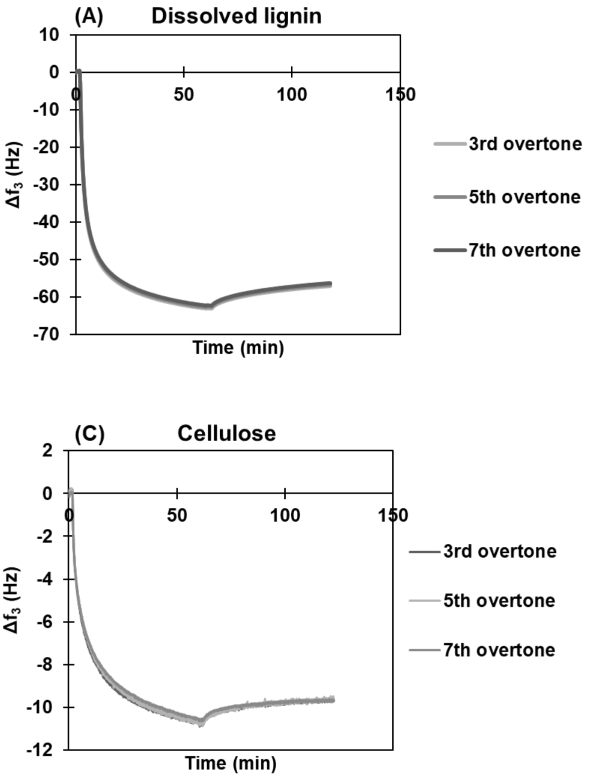

Figure S5. QCM-D detection of the frequency change of keratin onto (A) lignin, (B) CLP and (C) cellulose coated gold sensors for three different overtones (3rd, 5th, and 7th).

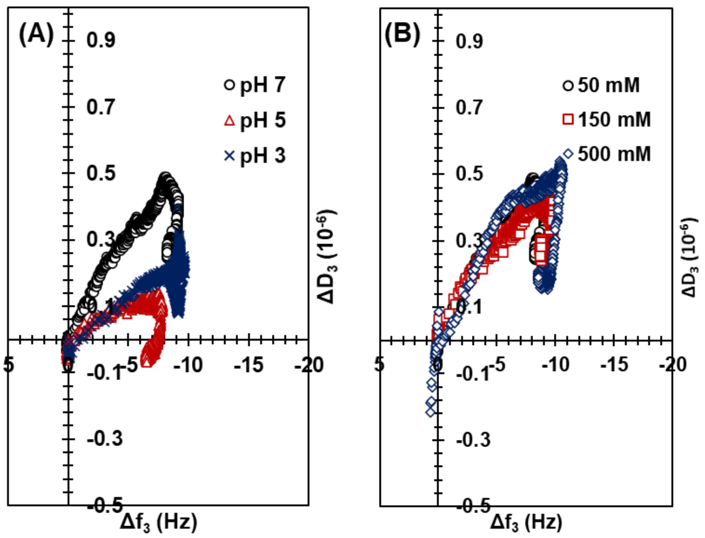

Figure S6. Change in dissipation factor as a function of the change in frequency for adsorption of keratin on cellulose at different (A) pHs and (B) ionic strengths.
